# Association between beliefs about medicines and self-medication with analgesics among patients with dental pain

**DOI:** 10.1371/journal.pone.0201776

**Published:** 2018-08-02

**Authors:** Piyush Mittal, Oi Yun Chan, Sham Kishor Kanneppady, Rohit Kumar Verma, Syed Shahzad Hasan

**Affiliations:** 1 Department of Pharmacy Practice, International Medical University, Kuala Lumpur, Malaysia; 2 School of Dentistry, International Medical University, Kuala Lumpur, Malaysia; 3 Department of Pharmacy, University of Huddersfield, Huddersfield, West Yorkshire, United Kingdom; 4 School of Biological Sciences and Pharmacy, University of Newcastle, Newcastle, New South Wales, Australia; Universita degli Studi Europea di Roma, ITALY

## Abstract

Self-medication with analgesics in dental pain management is a common practice as most of these medicines are available over-the-counter (OTC). The study aims to examine the relationship between beliefs about medicines and self-medication with analgesics in dental pain management in Malaysia. This cross-sectional study was conducted among conveniently sampled patients attending dental clinics, located in Kuala Lumpur, Malaysia to assess association between self-medication with analgesics and patient’s beliefs about medicines via Beliefs about Medicines Questionnaire. Participants were evaluated for their self-medication practices via 4 items. Further assessment was done via Quantitative Analgesic Questionnaire (QAQ) regarding the analgesics taken. Statistical analyses were performed using SPSS version 24, with 0.05 as level of significance. The prevalence of self-medication with analgesics was 29.4%, with 95.6% of the participants took analgesics when necessary. Participants practising self-medication for dental pain reported more positive beliefs in General-Necessity (13.04 vs. 9.98, p = 0.001) than those not practising self-medication. However, these participants had weaker beliefs in General-Harm (12.00 vs. 10.29, p = 0.006) and General-Overuse (11.38 vs. 10.31, p = 0.032) than those not practising self-medication. Participants beliefs in General-Harm (r = -0.243; p = 0.003) and General-Overuse (r = -0.203; p = 0.012) were negatively correlated with total QAQ point. The study found that individuals who practised self-medication had stronger beliefs about the benefits of medicines and weaker beliefs in viewing medicines as harmful and overused. Findings can guide public education to improve the safety aspects of self-medication with analgesics in dental practice.

## Introduction

Dental pain is a commonly reported oral health problem which can cause detrimental impact on normal physical health and psychological well-being [1–3]. In dental pain management, pain alleviation is of the utmost importance and analgesics including opioid and non-opioid analgesics are commonly prescribed to alleviate this pain [4–7]. Non-opioid analgesics including paracetamol and ibuprofen can be obtained without prescription in most of the countries including Malaysia. Thus, patients experiencing dental pain tend to self-medicate with these analgesics in hopes of avoiding dental treatment [1,8].

According to the World Health Organisation, self-medication is defined as the use of medicine for self-diagnosed disorders or the intermittent or continued use of a prescribed medicine for recurring or chronic disorders [9]. Currently, self-medication among patients with dental pain is a common practice driven by various underlying factors. Surprisingly, it was found that beliefs about medicines contributed to the practice of self-medication [1,8,10–13].

Belief is the mental acceptance of the actuality of an idea [14]. According to self-regulatory theory, the decision on choosing a coping action for an illness is influenced by beliefs about the potential cure of that illness [15]. Beliefs about medicines are commonly evaluated by Beliefs about Medicines Questionnaire (BMQ) [15]. BMQ can be used as the predictor of medication adherence. However, a recent study conducted in Sweden reported that individuals who used Over-The-Counter medicines had stronger beliefs about the benefits of medicines and weaker beliefs in viewing medicines as harmful and overused [16–20]. Thus, it was postulated that beliefs about medicines were related to self-medication as these beliefs might influence patient’s decision on taking a certain medicine for self-diagnosed disorders.

To the best of our knowledge, there is no information on the relationship between beliefs about medicines and self-medication with analgesics in patients with dental pain. As analgesic is the most frequently used medicine for self-medication in dental pain management and potential adverse consequences including renal failure can occur due to prolonged use of analgesics [1,8,10–12], the main objective of this study was to evaluate the relationship between beliefs about medicines and self-medication with analgesics in dental pain management.

## Methods

### Ethical approval

Ethical approval was obtained from IMU Joint Research and Ethics Committee [Research ID: BP I-01/14(57)2017]. Participants’ personal data were stored in a password-protected file accessible only to the researchers. No personal data was disclosed, and the study results are reported as de-identified data.

### Sample frame

This was a cross-sectional study conducted among adult patients attending dental clinics associated with Oral Health Centre (OHC) at International Medical University (IMU), Malaysia. Patients who met the inclusion criteria including aged 18 years and above, had dental pain, and those who had provided informed consent were recruited. Patients who did not meet these inclusion criteria were excluded. Though all patients with dental pain were invited to participate, a minimum sample size of 150 was estimated (distribution of response 50%, with 95% confidence interval and a 5% margin of error). A total of 170 patients was approached, but the final participating number was 153 which was more than the required sample size.

### Sampling procedure

The target population were sampled from dental clinics at International Medical University. The target population were interviewed in English. The study information sheets were given and written consent were obtained from each participant prior to participation in this study. Convenience sampling was used to recruit study participants attending OHC at IMU during the study period (July-September 2017). Data were collected via an interviewer-administered data collection form, assessing patient’s sociodemographic, dental pain-related characteristics, beliefs about medicines and use of analgesics for self-medication. Participant’s sociodemographic including age, gender, ethnicity, education, occupation, marital status, medical history and oral health-related characteristics was obtained.

### Dental pain-related characteristics

The modified dental pain questionnaire (M-DePaQ), a valid tool to classify dental pain was used to elicit participant’s dental pain-related characteristics including the pattern and location of pain [21,22]. In addition, participant’s dental pain intensity was measured with the single item scale ranging from 0 (no pain) to 5 (pains worst).

### Beliefs about Medicines Questionnaire (BMQ)

Participant’s beliefs about medicines were evaluated by using BMQ-Specific and BMQ-General [15]. Permission was obtained from the developer to use BMQ. Assessment of BMQ-Specific included subscales of Specific-Necessity (5 items) and Specific-Concern (5 items) while assessment of BMQ-General included subscales of General-Harm (4 items) and General-Overuse (4 items) [15].

All BMQ subscales were evaluated with a 5-point Likert scale of 1 (strongly disagree) to 5 (strongly agree). Points gained were summed up to generate total score for each subscale of BMQ-Specific (range: 5–25) and BMQ-General (range: 4–20) [15]. Higher scores gained for Specific-Necessity and Specific-Concern indicated participants had stronger beliefs in personal need and concern regarding analgesics, respectively. Meanwhile, higher scores obtained for General-Harm and General-Overuse indicated participants had higher beliefs in viewing medicines in general as harmful and being overused by dentists, respectively [15,17,18].

### Self-medication

Participants were evaluated for their self-medication practices via 4 items answered with “yes” or “no”. Further assessment was done via Quantitative Analgesic Questionnaire (QAQ) regarding the analgesics taken. QAQ is a validated questionnaire developed to record the actual use of analgesics as reported by patients [23]. Approval to use QAQ was obtained from the developer. Total QAQ point was calculated as shown in S1 Table. Participants who did not self-medicate with analgesics were given zero score while participants who took a higher total weekly dose of analgesic gained a higher total QAQ point [23].

### Statistical analyses

Statistical analyses were performed using Statistical Package for the Social Sciences (SPSS) version 24, with 0.05% as level of significance. The reliability of BMQ was tested with Cronbach’s alpha. Descriptive statistics were used to describe mean, standard deviation (SD), frequency and percentage. Independent t-test and Chi-squared test were done to determine the differences in mean scores of independent groups and to evaluate the association between categorical data, respectively. Meanwhile, the relationship between beliefs about medicines and total QAQ point was evaluated via Spearman’s correlation.

## Results

### Sociodemographic characteristics

A total of 153 patients participated in this study. Participant’s sociodemographic and dental pain intensity are presented in Table 1. The mean age of participants was 43.4 ± 15.2 (range: 19–75). Over half of the participants were female (58.8%) and approximately three-quarter of them were Chinese (71.9%). The mean dental pain intensity was 2.7 ± 1.2 (range: 1–5).

**Table 1:** Sociodemographic of participants (n = 153).

Items	n (%)
**Age** (mean ± SD)	43.4 ± 15.2
19–30	40 (26.1)
31–42	37 (24.2)
43–55	39 (25.5)
55–75	37 (24.2)
**Sex**	
Male	63 (41.2)
Female	90 (58.8)
**Race**	
Chinese	110 (71.9)
Indian	20 (13.1)
Malay	7 (4.6)
Others	16 (10.5)
**Education**	
Primary	5 (3.3)
Secondary	39 (25.5)
Diploma	33 (.6)
Graduation	76 (49.7)
**Long standing illness**	
Yes	45 (29.4)
No	108 (70.6)
**Overall health**	
Excellent	13 (8.5)
Good	96 (62.7)
Fair	41 (26.8)
Bad	3 (2.0)
**Pain intensity**, mean ± SD (range)	2.7 ± 1.2 (1–5)

### Dental pain-related characteristics

Participant's dental pain-related characteristics are reported in Table 2. Most participants (79.1%) experienced pain originating from their tooth/teeth in the past one month. Majority of the participants had mild (57%) and episodic (77%) dental pain.

**Table 2:** Frequency distribution of the modified dental pain questionnaire (n = 153).

Items	Frequency (n)	Percentage (%)
**Pain in the** ..		
Tooth/teeth	121	79.1
Gums	73	47.7
Both	28	18.4
**Pain for** .. (chronicity of current pain)		
Less than 1 week	37	24.2
From 1 week or longer	116	75.8
**Pain is** .. (dental pain intensity)		
Mild	72	56.9
Discomforting and distressing	38	24.8
Horrible and excruciating	43	28.1
**Pain has been**…(pattern of current dental pain)		
Episodic	118	77.1
Continuous	35	22.9
**Pain radiates to the surrounding area**		
No	103	67.3
Yes	50	32.7
**Pain worse when chewing and eating**		
Yes	115	75.2
No	38	24.8
**Eating or drinking something cold makes pain worse**		
Yes	77	50.3
No	76	49.7
**Gums swollen**		
Yes	60	39.2
No	93	60.8
**Painful tooth feels like it is loose**		
Yes	43	28.1
No	110	71.9
**Difficulty to swallow**		
Yes	17	11.1
No	136	88.9
**Painful tooth feels like it is sticking out**		
Yes	32	20.9
No	121	79.1
**Difficulty in sleeping**		
Yes	58	37.9
No	95	62.1

### Beliefs about medicines

BMQ-Specific and BMQ-General subscales demonstrated good and acceptable internal consistency, respectively (Specific-Necessity, α = 0.781; Specific-Concern, α = 0.770; General-Harm, α = 0.620; General-Overuse, α = 0.520). The mean score of each item of BMQ was reported in Table 3. Meanwhile, the mean total score of Specific-Necessity, Specific-Concern, General-Harm and General-Overuse were 10.9 ± 4.5 (range: 5–22), 13.9 ± 5.1 (range: 5–25), 11.5 ± 3.5 (range: 4–20) and 11.1 ± 2.8 (range: 4–20), respectively.

**Table 3:** Mean score for each item of BMQ subscale (n = 153).

Item	Mean	SD	Min.	Max.
**Specific-Necessity**				
My oral health, at present, depends on my medicines for dental pain.	1.9	1.2	1	5
My life would be impossible without my medicines for dental pain.	2.2	1.4	1	5
Without my medicines for dental pain I would be very ill.	2.2	1.3	1	5
My oral health in the future will depend on my medicines for dental pain.	1.8	0.9	1	4
My medicines for dental pain protect me from becoming worse.	2.8	1.3	1	5
**Specific-Concern**				
Having to take medicines for dental pain worries me.	2.9	1.5	1	5
I sometimes worry about the long-term effects of my medicines for dental pain.	3.5	1.5	1	5
My medicines for dental pain are a mystery to me.	2.5	1.3	1	5
My medicines for dental pain disrupt my life.	2.2	1.3	1	5
I sometimes worry about becoming too dependent on my medicines for dental pain.	2.7	1.5	1	5
**General-Harm**				
People who take medicines should stop their treatment for a while every now and again.	2.9	1.3	1	5
Most medicines are addictive.	2.6	1.3	1	5
Medicines do more harm than good.	3.2	1.2	1	5
All medicines are poisons.	2.8	1.3	1	5
**General-Overuse**				
Dentists use too many medicines.	1.9	1.0	1	5
Natural remedies are safer than medicines.	3.8	1.2	1	5
Dentists place too much trust in medicines.	2.4	1.0	1	5
If dentists had more time with patients, they would prescribe fewer medicines.	2.9	1.2	1	5

Note: BMQ adopted as developed and validated by Horne et. al.[15]

### Self-medication and beliefs about medicines

Based on Table 4, the prevalence of self-medication with analgesics was 29.4%, with 95.6% of the participants took analgesics when necessary. Paracetamol was (42.2%) reported as the most frequently used analgesic followed by ibuprofen (15.6%) and mefenamic acid (13.3%) for self-medication in dental pain management. Meanwhile, the mean total QAQ point was 0.4 ± 0.8 (range: 0–4).

**Table 4:** Frequency distribution of the items assessing self-medication.

Item	Frequency (n)	Percentage (%)
Self-medication with analgesics (n = 153)		
Yes	45	29.4
No	108	70.6
Analgesic taken regularly* (n = 45)		
Yes	2	4.4
No	43	95.6
Analgesic taken when necessary* (n = 45)		
Yes	43	95.6
No	2	4.4
Analgesic taken relieved pain* (n = 45)		
Yes	34	75.6
No	11	24.4

* Items answered only by participants who self-medicated with analgesics.

Table 5 presents mean scores of BMQ subscales among participants practising self-medication and those not practising self-medication. Participants practising self-medication with analgesics for dental pain reported more positive beliefs in Specific-Necessity (13.0 vs. 9.9, p = 0.001) compared with those not practising self-medication. On the other hand, participants not practising self-medication reported more positive beliefs in General-Harm (p = 0.006) and General-Overuse (p = 0.032) as compared with participants practising self-medication. However, no statistically significant difference was noted in Specific-Concern (p = 0.916).

**Table 5:** Mean total score of BMQ subscales among participants who practised self-medication and those who did not.

Item	Specific-Necessity		Specific-Concern		General-Harm		General-Overuse	
	Mean ± SD	p-value	Mean ± SD	p-value	Mean ± SD	p-value	Mean ± SD	p-value
Self-medication with analgesics (n = 153)								
Yes	13.0±4.9	**0.001**	14.0±5.6	0.916	10.3±3.2	**0.006**	10.3±3.0	**0.032**
No	9.9±4.1		13.9 ±4.9		12.0 ±3.6		11.4 ±2.7	
Analgesic taken regularly (n = 45)								
Yes	19.5 ±3.5	0.058	16.5 ±4.9	0.526	9.5 ±3.5	0.726	8.5 ±3.5	0.396
No	12.7 ±4.8		13.9 ±5.6		10.3 ±3.2		10.4 ±3.0	
Analgesic taken when necessary (n = 45)								
Yes	12.7 ±4.8	0.058	13.9 ±5.6	0.526	10.3 ±3.2	0.726	10.4 ±3.0	0.396
No	19.5 ±3.5		16.5 ±4.9		9.5 ±3.5		8.5 ±3.5	
Analgesic taken relieved pain (n = 45)								
Yes	13.3 ±4.9	0.469	13.5 ±5.5	0.247	10.3 ±3.4	0.900	10.4 ±3.3	0.701
No	12.1 ±5.3		15.7 ±5.6		10.2 ±2.4		10.0 ±2.1	

Note: Mean values were compared and p-values were determined via independent t-test.

There was a significant positive correlation between beliefs in Specific-Necessity and total QAQ point (r = 0.290; p < 0.001). Meanwhile, beliefs in General-Harm (r = -0.243; p = 0.003) and General-Overuse (r = -0.203; p = 0.012) were negatively correlated with total QAQ point. No correlation between was found between Specific-Concern and total QAQ point.

## Discussion

The present study attempted to investigate the prevalence of self-medication with analgesics and to provide a further insight on the relationship between beliefs about medicines and self-medication for dental pain. It was reported in this study that slightly more than one-quarter of the participants (29.4%) practised self-medication with analgesics. The prevalence found in this study is similar to the prevalence of self-medication (30%) reported in previous study by Simon et al., [10], and lower prevalence than a study by Jain et al., [11], where approximately three-quarter of the participants (72%) practised self-medication.

This finding supported the idea suggested by previous studies that the difference in prevalence of self-medication was attributed to the dissimilar clinical settings [10,11]. Recruited participants in this study were patients attending OHC. Similarly, patients presenting at oral health outreach programs were recruited in the study conducted by Simon et al., [10]. In contrast, the study conducted by Jain et. al. targeted customers visiting pharmacies [11]. The lower prevalence reported in this study and the study conducted by Simon et al., may be explained by the fact that not all oral health problems were eventually brought to the dentist’s attention as patients might experience pain relief after self-medicating with analgesics obtained from pharmacies [10–12].

Results indicate that participants practising self-medication with analgesics for dental pain reported more positive beliefs in personal need for analgesics and did not view medicines in general as harmful and overused. The present study also found a significant positive correlation between beliefs in Specific-Necessity and total QAQ point. This indicates that as the participants beliefs in personal need and concern regarding analgesics become stronger, they take a higher total weekly dose of analgesic. Similarly, as participants beliefs in viewing medicines in general as harmful and being overused become stronger, they take a lower total weekly dose of analgesic.

These results were similar to a previous study by Sundell et al., [20], who assessed BMQ-General among the general population in relation to the use of Over-The-Counter medicines. According to Sundell et. al., individuals who took Over-The-Counter medicines had higher beliefs in the benefits of medicines but lower beliefs in viewing medicines as harmful and overused [20].

In view of these findings, the association between self-medication and beliefs in Specific-Necessity, General-Harm and General-Overuse could be attributed to the fact that patients with high positive beliefs about analgesics (Specific-Necessity) and low negative beliefs about medicines (General-Harm and General-Overuse) tend to perceive analgesic as a potential cure to dental pain. These beliefs lead to their decision to choose self-medication with analgesics as a coping action for dental pain [14,15].

However, beliefs in Specific-Concern was not significantly associated with self-medication and total QAQ score although it was considered as one of the negative beliefs. Patients with stronger beliefs in Specific-Necessity may recognise the potential benefits and role of analgesics in protecting their present and future oral health against deterioration. Moreover, nearly all patients in this study who practised self-medication took analgesics only when necessary. This suggest that they self-medicate with analgesics regardless of their concerns about the potential adverse consequences of taking analgesics such as the long term effects or worried of becoming too dependent on analgesics [14,15]. Another reason could be mild and episodic nature of dental pain as reported by majority of the study participants.

Paracetamol (also known as acetaminophen) was the most commonly used analgesic by patients with dental pain in this study. Paracetamol decreases acute dental pain [24] and is commonly used by patients who have dental pain [25–27]. The problem of overdose with paracetamol is frequently reported by previous studies [25–27]. In a recent study by O’Sullivan et al (2018) found that 38% of overdose patients treated in accident and emergency departments are caused by dental pain [25]. We recommend that dentists should refer to relevant guidelines on prescribing analgesics for dental pain [28,29]. No doubt the practice of self-medication provides benefits but it is also associated with potential risks including drug-drug interactions and misdiagnosis that can lead to severe complications [30,31]. Therefore, dentists should consider taking complete medication history including self-medication.

## Strengths and limitations

There were some limitations in this study that need to be addressed and taken into consideration while interpreting the results. Firstly, there might be recall bias as subjective instrument was used to assess patient’s actual use of analgesics. In addition, non-probability sampling used might lead to selection bias among participants. Moreover, BMQ-General showed poor internal consistency which was probably due to the small number of items included. Furthermore, this was a single-centre study where the target population was limited to patients attending OHC. Thus, the results obtained might not be a good representative of a larger population.

Despite these limitations, there were some strengths in the present study. To the best of our knowledge, this was the first study conducted in Malaysia assessing the relationship between beliefs about medicines and self-medication with analgesics in dental pain management. Besides that, data was collected via interviewer-administered data collection form. Utilising an interviewer-administered data collection form is of substantial importance to minimise potential bias as participants were given the opportunity to clarify any doubts [32].

## Conclusions

The study found that adult patients attending Oral Health centre at International Medical University, who practised self-medication had stronger beliefs about the benefits of medicines and weaker beliefs in viewing medicines as harmful and overused. No significant difference between those practising self-medication and those not practising in terms of beliefs in Specific-Concern. It is crucial to confirm whether beliefs about medicines are associated with self-medication as these findings can provide guidelines in public education to improve the safety aspects of self-medication. Upon considering the strengths and limitations, future research with relevant modifications such as targeting a larger population in different clinical settings is certainly needed to further confirm the results reported.

## Supporting information

S1 Table Sample calculation for QAQ points. (DOCX)
