# Supplementary material for: Association between beliefs about medicines and self-medication with analgesics among patients with dental pain
Source: PLoS One. 2018 Aug 2;13(8):e0201776. doi: 10.1371/journal.pone.0201776 (PMC6072109; doi:10.1371/journal.pone.0201776)
Supplement: S1 Table — (DOCX) [file pone.0201776.s001.docx]

S1 Table – Sample calculation for QAQ points

Sample calculation for QAQ points.[23]

| **Medication** | **Dose (mg)** | **Days per week** | **Pills per day** | **Total weekly dose (mg)** | **Morphine equivalent of weekly dose (for opioids only)** | **% of maximum dose* (for non-opioids)** | **QAQ point** |
| --- | --- | --- | --- | --- | --- | --- | --- |
| Paracetamol | 500 | 2 | 1 | 500 x 2 x 1  = 1000 | - | $\frac{1000}{4000 x 7} x 100\%=3.57\%$ | 1 |
| Ibuprofen | 400 | 2 | 3 | 400 x 2 x 3  = 2400 | - | $\frac{2400}{1200 x 7} x 100\%=28.57\%$ | 2 |
| Codeine | 8 | 2 | 1 | 8 x 2 x 1  = 16 | 16 mg x 0.15 =  2.4 mg | - | 1 |
|  |  |  |  |  |  | Total QAQ Point | 4 |

Note: The total weekly dose was calculated (dose x days per week x pills per day). Next, the total weekly dose for opioid analgesics were converted into equivalent mg of oral morphine and summed. Meanwhile, the total weekly dose for all other non-opioid analgesics were converted into percentage of maximum recommended dose. For opioid analgesics, one point was assigned for taking any opioid analgesics at all and an additional point was assigned for every equivalent of 100mg of oral morphine used (e.g., 1-99mg = 1 point; 100-199mg = 2 points, etc.). For non-opioid analgesics, one point was assigned for taking the medication at all and an additional point was assigned for each 25% of the maximum dose taken (e.g., 1-24% = 1 point; 25-49% = 2 points, etc.). Lastly, the total QAQ point was calculated by adding each QAQ point gained for each type of analgesics taken.[23]
